# Mobile triage units for abdominal trauma in sub-Saharan Africa: current evidence, implementation barriers, and strategies for scale-up

**DOI:** 10.1097/MS9.0000000000004655

**Published:** 2025-12-26

**Authors:** Temitomi Jane Oyedele, Olamide Funbi Ogunbodede, Adiat Chinonye Oyeneyin, Nifemi Tunmise Odunsi, Tolulope Oluwafikayo Awotunde, Adepeju Anuoluwa Agunbiade, Nicholas Aderinto

**Affiliations:** a Department of Medicine and Surgery, Bowen University, Iwo, Nigeria; b Department of Medicine and Surgery, Ladoke Akintola University of Technology, Ogbomoso, Nigeria

**Keywords:** abdominal trauma, mobile triage units, prehospital emergency care, sub-Saharan Africa, trauma systems

## Abstract

Abdominal trauma is a major contributor to morbidity and mortality in sub-Saharan Africa (SSA), exacerbated by systemic deficiencies in prehospital care, limited hospital resources, and reliance on informal transport. Mobile triage units (MTUs) have emerged as a potential solution, providing rapid on-scene assessment, stabilization, and diagnostics. This narrative review synthesized evidence from PubMed, African Journals Online, Embase, Scopus, Web of Science, and Google Scholar to evaluate MTU roles, effectiveness, implementation challenges, and strategies for sustainable deployment in SSA. Trauma-related deaths are substantial, with up to 50% occurring in the prehospital phase. MTUs equipped with portable ultrasound, hemorrhage control tools, fluid resuscitation kits, telemedicine, and drone-assisted supply delivery demonstrate the potential to reduce prehospital mortality by up to 30% and decrease transport times. Task-shifting models and community first responder programs enhance MTU reach in resource-limited areas. Implementation challenges include financial constraints, workforce shortages, logistical barriers, integration difficulties, and cultural acceptance. Addressing these requires targeted strategies: cost-effective and locally adapted designs to reduce financial barriers; scalable training programs and task-shifting to address workforce gaps; mobile and drone-assisted units to overcome logistical challenges; EMS integration to ensure coordinated care; and community engagement to foster acceptance. MTUs represent a transformative intervention for SSA, improving prehospital trauma care and timely access to definitive management. Further multicenter studies are needed to evaluate effectiveness, optimize operational protocols, and identify best practices for scalable MTU deployment across the region.

## Introduction

Trauma remains a major global public health challenge, responsible for an estimated 4.4 million deaths annually and accounting for 8% of all deaths worldwide, with nearly 18% occurring in Africa^[1,2]^. Abdominal trauma, defined as injury to the intra-abdominal organs from blunt or penetrating mechanisms, represents a particularly severe subset, contributing 7 to 10% of all trauma-related fatalities globally^[3]^. In sub-Saharan Africa (SSA), mortality from abdominal trauma varies widely from 2% to 28%, reflecting disparities in healthcare infrastructure, timely access to surgical care, and prehospital management^[3]^. In Tanzania, for example, abdominal trauma accounts for 24% of trauma admissions, with a mortality rate of 13.3%^[4]^. The leading etiologies remain road traffic collisions, followed by interpersonal violence, falls, and conflict-related injuries^[5]^.

**HIGHLIGHTS**

- Abdominal trauma contributes to 7–10% of trauma-related fatalities in sub-Saharan Africa (SSA).
- Mobile triage units (MTUs), equipped with tools like point-of-care ultrasound and telemedicine, reduce prehospital mortality by up to 30% and transport times by up to 75 min in SSA pilot programs, addressing critical gaps in triage and stabilization.
- Financial constraints, workforce shortages, and logistical barriers hinder MTU scalability, necessitating cost-effective designs, scalable training, public–private partnerships, and policy integration for sustainable deployment.

Early prehospital assessment and stabilization are critical determinants of survival in abdominal trauma^[6]^. However, most SSA countries lack organized emergency medical services (EMS), resulting in substantial prehospital delays that contribute to preventable mortality^[7]^. Only 10 to 15% of trauma patients in the region receive any form of prehospital care^[8]^. In Rwanda, nearly half of trauma-related deaths occurred within 24 h of hospital arrival, underscoring systemic inefficiencies across the trauma care continuum^[9]^. According to the World Health Organization’s Trauma System Maturity Index, the majority of SSA countries rank poorly in prehospital organization, trauma center availability, personnel training, and quality assurance, with South Africa being the only country classified at Level III maturity^[10]^. Structural barriers, including limited infrastructure, scarcity of trauma centers, shortage of trained personnel, weak EMS policies, and chronic underfunding, further delay triage and definitive management, leading to adverse outcomes in abdominal trauma^[11]^.

Triage, the systematic categorization of patients by injury severity to prioritize life-saving interventions, is particularly vital in resource-constrained settings^[12]^. Mobile triage units (MTUs) have been introduced as a decentralized, context-adapted solution to address these barriers^[13]^. MTUs are mobile platforms, often vehicle- or motorcycle-based, equipped to perform on-scene triage, stabilization, and rapid transfer coordination. By bypassing infrastructure limitations, they provide timely assessment; through task-shifting, they mitigate personnel shortages; and by incorporating portable diagnostic tools such as Focused Assessment with Sonography for Trauma (FAST), they enable early detection of life-threatening injuries and faster referral to definitive care^[14]^.

This paper reviews the role of MTUs in the rapid assessment and stabilization of abdominal trauma in SSA. It further explores current innovations, implementation challenges, and strategic opportunities for sustainable integration within regional trauma systems. The study also adheres to the Transparency in the Reporting of Artificial Intelligence (TITAN) guidelines on the use of artificial intelligence in scientific writing^[15]^.

## Methodology

### Study design

This study employed a narrative review design to synthesize existing evidence on the use of MTUs for the rapid assessment and stabilization of abdominal trauma in SSA. A narrative approach was selected to integrate findings from diverse study designs and program evaluations.

### Literature search strategy

A literature search was conducted in PubMed, African Journals Online (AJOL), Embase, Scopus, Web of Science, and Google Scholar, covering all available years from database inception through July 2025.The search combined Medical Subject Headings (MeSH) and free-text terms related to abdominal trauma and prehospital triage models. The core Boolean structure used was: (“abdominal trauma” OR “abdominal injury” OR “blunt abdominal trauma”) AND (“mobile triage unit” OR “mobile emergency unit” OR “prehospital triage” OR “emergency medical services” OR “mobile health unit”) AND (“Sub-Saharan Africa” OR “Africa South of the Sahara”). Supplemental Digital Content File 1, available at: http://links.lww.com/MS9/B69. Reference lists of all included studies and relevant reviews were also screened to identify additional eligible publications.

### Eligibility

#### Inclusion criteria

Studies were included if they met all the following criteria:

- Focused on abdominal trauma and/or prehospital trauma care in SSA.

- Evaluated MTUs or prehospital innovations directly aimed at improving triage, stabilization, or rapid transport of trauma patients. Relevant innovations were operationally defined as interventions or technologies that:
  1. Enhance on-scene assessment, e.g., portable diagnostic tools such as FAST.
  2. Improve resuscitation or stabilization, e.g., portable fluid kits, hemorrhage control devices.
  3. Facilitate rapid or safe patient transport, e.g., motorcycle-based MTUs, ambulance modifications, or drone-assisted delivery of blood products or essential supplies.
  4. Strengthen communication and decision-making, e.g., telemedicine for remote guidance by trained clinicians.

- Reported empirical data, policy frameworks, or program evaluations from peer-reviewed articles.

#### Exclusion criteria

- Non-English language publications without accessible translation.
- Commentaries, editorials, or opinion pieces lacking empirical data or policy/program evaluation content.
- Innovations not directly linked to prehospital trauma care (e.g., general hospital management technologies, non-trauma health interventions).

### Study selection and data extraction

All retrieved titles and abstracts were screened for relevance by two independent reviewers [NA and TMJ], followed by full-text assessment. Discrepancies were resolved through discussion and consensus. Extracted studies were then grouped into thematic domains for a narrative synthesis examining the global context, current state of MTU in Africa, barriers to implementation, and solutions.

### Synthesis approach

Given the methodological and contextual heterogeneity of included studies, a narrative synthesis was undertaken. Evidence was summarized descriptively to highlight epidemiologic patterns, operational barriers, and the potential contribution of MTUs and related innovations to reduce preventable trauma deaths in SSA. Where appropriate, cross-cutting themes were identified to inform future implementation strategies and policy development.

## Global and regional context of prehospital care

Prehospital care represents a cornerstone of modern trauma systems, designed to deliver rapid assessment, stabilization, and transport that optimize outcomes for injured patients, particularly those with abdominal trauma^[8]^. In SSA, where trauma contributes substantially to morbidity and mortality, the lack of structured prehospital systems worsens outcomes up to 50% of trauma-related deaths occurring before hospital arrival^[16]^. MTUs have emerged as a novel solution to address these gaps. An MTU is a mobile, often vehicle-based platform equipped to provide rapid on-scene triage, resuscitation, and coordination of transport to definitive care^[17,18]^. The concept of MTUs was first developed in high-income countries (HICs) to improve prehospital response times in rural or resource-limited settings, drawing on innovations from mobile emergency care, ambulance services, and disaster response units^[17]^. Over time, MTUs have evolved to include portable diagnostic tools, such as FAST, point-of-care laboratory tests, hemorrhage control kits, airway management equipment, and telemedicine capabilities to connect with specialist clinicians remotely^[18]^.

### Prehospital care in HICs

In HICs, EMS are integral to organized trauma systems, operating within regulated and coordinated healthcare frameworks^[19]^. These systems ensure seamless care from the site of injury to definitive management, resulting in measurable reductions in trauma mortality and morbidity^[20,21]^. A defining strength of HIC EMS lies in structured triage protocols that prioritize patients by physiological severity and injury mechanism. The Rapid Emergency Triage and Treatment System (RETTS), for instance, employs a five-tier color-coded framework based on vital signs and clinical presentation to enable rapid identification of critical cases^[22]^. Similarly, in the United States, the Emergency Severity Index (ESI) and the American College of Surgeons’ Field Triage Guidelines provide standardized criteria that emphasize physiological parameters (blood pressure, respiratory rate), anatomical patterns (penetrating abdominal injuries), and mechanisms of injury (high-energy collisions) to direct patients to appropriate trauma centers^[23,24]^. Such systems permit EMS providers to bypass lower-level facilities, ensuring patients with severe abdominal trauma receive timely surgical care^[25]^.

The infrastructure in HIC EMS is advanced. Ambulances are equipped with comprehensive life-support capabilities, including airway management tools (endotracheal intubation), intravenous access and resuscitation kits, blood transfusion capacity, and point-of-care ultrasound (POCUS) for rapid diagnosis^[26]^. These units are staffed by highly trained paramedics, EMTs, and, in some regions, emergency physicians capable of performing complex field interventions. Additionally, prehospital notification systems allow hospitals to mobilize trauma teams in advance, reducing time to operative management^[27]^. In the United Kingdom, for example, prehospital notification has been shown to shorten time to laparotomy for abdominal trauma by approximately 30%, thereby improving survival^[28]^.

The impact of these systems is well documented. Evidence from Europe and North America indicates that structured EMS frameworks reduce prehospital mortality by up to 25%, expedite definitive care, and have contributed to a sustained decline in trauma-related deaths over recent decades^[29,30]^. For abdominal trauma, survival rates are enhanced by early stabilization and timely surgical intervention within the “golden hour,” when prompt management maximizes outcomes^[31]^. These achievements reflect not only in-hospital expertise but also the prehospital system’s ability to prevent physiological deterioration during transport. See Table 1.

**Table 1:** Comparison of prehospital care systems in high-income countries and sub-Saharan Africa

Aspect	High-income countries	Sub-Saharan Africa	Source
EMS Organization	Highly organized, regulated, and integrated into healthcare systems	Fragmented or absent, limited to urban areas	^[19,32]^
Triage protocols	Structured (e.g., RETTS, ESI, ACS Field Triage Guidelines)	Often absent; limited use of SATS in some areas	^[22,23,33]^
Transport	Well-equipped ambulances with ALS capabilities	Informal (taxis, motorbikes, private cars); ambulances lack equipment	^[26,34,35]^
Interventions	Airway management, IV resuscitation, POCUS, blood transfusion	Minimal or absent; basic first-aid in some cases	^[26,36]^
Time to care	Within “golden hour” (often <1 h)	Delays of 6–12 h; only 24.4% reach specialists in 6 h (Nigeria)	^[18,37,38]^
Prehospital mortality	Low (5–10% for abdominal trauma)	High (up to 50% for trauma)	^[31,39]^
Outcomes	25% Reduction in mortality due to EMS	High mortality due to a lack of stabilization	^[29,40]^

### Prehospital care in SSA

In contrast, prehospital care in SSA remains fragmented, under-resourced, and, in many regions, entirely absent, contributing directly to preventable trauma deaths. Formal EMS systems are generally confined to major cities, leaving vast rural populations without organized emergency transport^[32]^. Most trauma victims rely on informal transportation such as police vehicles, taxis, motorcycles, or private cars, which lack trained personnel, resuscitative capacity, or medical supplies^[34,35]^. Consequently, many patients arrive at hospitals without stabilization, a factor strongly associated with higher mortality^[36]^. In Ethiopia, for example, a single-center study reported that fewer than 10% of trauma patients received any prehospital intervention, with abdominal trauma mortality exceeding 40% in the prehospital phase^[37]^.

Systemic delays are a persistent challenge across SSA. In rural southeast Nigeria, only 24.4% of patients with penetrating abdominal trauma reached surgical care within 6 h of injury; delays were associated with a 35% rise in postoperative mortality^[38]^. Comparable patterns are reported in Tanzania, Ethiopia, and Sudan, where poor transport networks, limited communication, and multistage inter-facility transfers contribute to critical time loss^[41–43]^. In Tanzania, for instance, abdominal trauma patients often face transfers across multiple facilities, leading to surgical delays exceeding 12 h^[41]^. Such delays are particularly catastrophic in hemorrhagic abdominal injuries, where rapid decompensation can occur within minutes.

Even where formal EMS exists, its capabilities are frequently limited. The Lagos State Ambulance Service (LASAMBUS) in Nigeria, one of the most advanced in the region, illustrates this constraint. Evaluations reveal that most trauma patients arrive at Lagos State University Teaching Hospital without essential prehospital procedures such as airway management or intravenous access, reflecting deficits in training and equipment^[44]^. Similarly, in Morocco, only 15% of ambulances were equipped for advanced resuscitation, functioning primarily as basic transport vehicles^[45]^.

The consequences of these deficiencies are severe: up to half of trauma-related deaths in SSA occur before hospital arrival, and abdominal trauma patients are particularly vulnerable due to rapid progression to hemorrhagic shock^[39,40]^. The absence of structured triage, early resuscitation, and safe transfer mechanisms reinforces the urgent need for scalable, context-adapted innovations such as MTUs to bridge prehospital care gaps and improve trauma outcomes across the region.

## Burden of abdominal trauma in SSA

Abdominal trauma remains a major but under-recognized public health challenge in SSA, driven by rapid urbanization, increased motorization, and interpersonal violence^[46]^. Road traffic crashes, occupational hazards, and assault-related injuries are leading causes, collectively placing severe pressure on already constrained emergency and surgical care systems. Both penetrating and blunt mechanisms contribute substantially to this burden: penetrating trauma, often due to stabbings and gunshot wounds, predominates in urban centers, whereas blunt abdominal trauma from road traffic or occupational injuries is common across rural and peri-urban settings^[47]^. In Southeast Nigeria, penetrating abdominal injuries accounted for over 60% of operative cases^[48]^, while a study from Kenya demonstrates that blunt trauma (32.4%) comprises a significant proportion of trauma admissions^[49]^.

Mortality from abdominal trauma across SSA remains alarmingly high, frequently resulting from hemorrhagic shock, multiple-organ failure, and sepsis^[47,50]^. Prehospital deaths constitute a particularly large fraction of these losses. In Sudan, nearly 40% of abdominal trauma patients died before reaching a hospital, largely due to delayed transport and lack of early stabilization, which is particularly lethal in abdominal trauma because uncontrolled hemorrhage can rapidly lead to irreversible shock and death^[51]^. Such findings emphasize that timely prehospital assessment and intervention are critical determinants of survival. Yet, delays in care remain pervasive: in Southeast Nigeria, only 25% of patients with penetrating abdominal trauma were evaluated by a surgical specialist within 6 h of injury, with late presentation associated with a markedly higher postoperative mortality rate^[52]^. Similar patterns have been documented in Tanzania, Ethiopia, and Sudan, where poor road networks, limited communication systems, and reliance on sequential inter-facility referrals extend preoperative delays beyond 12 h in many cases^[53]^.

Prehospital systems in most SSA settings remain rudimentary or absent. Patients are often transported via informal means without trained personnel or resuscitative equipment^[54]^. Consequently, essential life-saving interventions such as hemorrhage control, airway protection, and fluid resuscitation are rarely administered, contributing to preventable deaths. In Ethiopia, fewer than 10% of trauma patients received any prehospital care^[55]^. The absence of structured emergency transport and stabilization capacity shows the urgent need for mobile, adaptable prehospital platforms such as MTUs, which can bridge gaps between community injury sites and definitive hospital care^[55]^.

Even after arrival at healthcare facilities, outcomes are compromised by limited diagnostic and operative capabilities. Advanced imaging modalities, including ultrasound and computed tomography, are often unavailable, compelling reliance on clinical assessment that may miss internal injuries^[56,57]^. Surgical readiness is equally constrained: many hospitals lack adequately trained surgeons, functioning operating theaters, blood products, and postoperative critical care capacity. In rural Tanzania, only a minority of hospitals can perform emergency laparotomies, and insufficient anesthetic and intensive care resources further elevate postoperative mortality^[58]^.

## Role of MTUs in SSA

MTUs are portable or vehicle-based platforms designed to provide prehospital triage, stabilization, and rapid transport for trauma patients in resource-limited settings^[59,60]^. MTUs were originally developed to bridge gaps in formal EMS, particularly in regions with limited coverage, high prehospital delays, and preventable trauma mortality^[8]^. Core components include portable ultrasound devices for FAST, airway management tools (bag-valve masks, endotracheal tubes), hemorrhage control supplies (tourniquets, hemostatic agents), and intravenous fluid kits^[60]^. Advanced units may incorporate telemedicine systems for real-time consultation with surgical specialists and drone-assisted supply delivery to overcome logistical barriers in remote areas^[61]^.

The primary function of MTUs is rapid triage using structured protocols, such as the ESI or the South African Triage Scale (SATS)^[33,62]^. These frameworks enable personnel to categorize patients into critical, urgent, or stable groups, ensuring that life-threatening abdominal injuries, including hemoperitoneum or visceral rupture, receive prioritized transport to surgical centers^[63]^. FAST ultrasound is central to this process, allowing early detection of intra-abdominal bleeding, guiding fluid resuscitation, and reducing the risk of hemorrhagic shock during transport^[63]^.

Staffing models reflect the realities of limited professional EMS personnel in SSA. MTUs are typically staffed by paramedics, EMTs, or trained community health workers, with task-shifting approaches training lay responders to perform basic interventions under remote supervision via telemedicine^[64]^. This flexibility enables MTUs to operate in both urban centers and hard-to-reach rural areas where conventional ambulances may be impractical^[65]^.

Emerging evidence demonstrates MTUs’ potential to improve outcomes for abdominal trauma. In Uganda, a pilot MTU program with portable ultrasound and resuscitation tools reduced prehospital mortality by 30% compared with standard transport^[66]^. In Rwanda, motorcycle-based MTUs staffed by trained community responders reduced median hospital arrival time from 120 to 45 min, improving survival^[67]^. Experiences from other LMICs, including India and South Africa, highlight scalable strategies such as mobile trauma units, reducing time to surgical intervention by 40% and decreasing abdominal trauma mortality from 25% to 15%^[68,69]^.

Innovations, including POCUS telemedicine and AI-assisted decision support, further enhance MTU effectiveness. AI algorithms applied to POCUS can detect intra-abdominal bleeding in real time, guiding early resuscitation and triage^[70–73]^. Predictive AI models may optimize patient prioritization and workflow, particularly in settings with limited trained personnel. Drone-assisted supply delivery ensures the timely provision of blood products or hemostatic agents to remote MTUs, addressing logistical barriers^[73–75]^.

The effectiveness of MTUs aligns with the “golden hour” principle, emphasizing rapid intervention within the first hour post-injury to maximize survival^[18]^. By providing early assessment, stabilization, and expedited transport, MTUs mitigate physiological deterioration associated with delays, particularly in abdominal trauma patients at high risk of hemorrhagic shock^[70,76]^.

**Figure 1.**
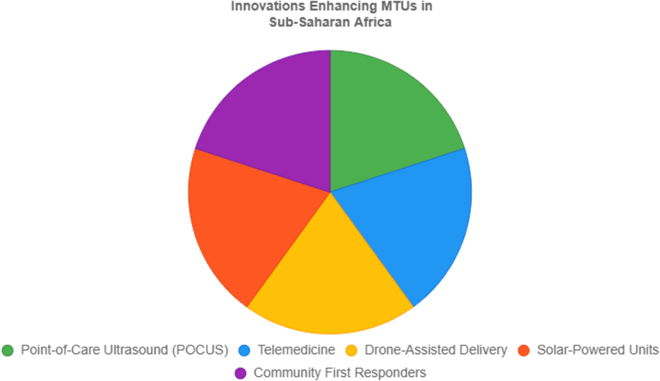
Innovations enhancing MTUs in SSA.

While encouraging, the current evidence base in SSA remains limited, with most data derived from pilot or single-center studies^[66–69]^. Larger, multicenter trials are needed to validate MTU impact, optimize staffing and equipment models, and assess cost-effectiveness for regional scale-up (Fig. 1).

## Implementation challenges

### Financial constraints

The high cost of equipping and maintaining MTUs is a major barrier in SSA. A fully equipped MTU is estimated to require an initial investment of approximately US$50 000–$100 000, including the vehicle, portable diagnostic equipment, hemorrhage control and resuscitation supplies, basic communication tools, and initial staff training^[77]^. To illustrate the magnitude of this cost, the annual per capita health expenditure in many SSA countries is below US$50, and the average monthly salary of a healthcare worker in Tanzania is approximately US$200–300^[78,79]^. Funding often relies on external donors, such as international NGOs or global health organizations, raising concerns about long-term sustainability^[74–76,80]^. Some pilot programs have explored low-cost or modular MTU designs using locally sourced materials, though large-scale feasibility evidence remains limited^[76,80]^ (see Table 2).

**Table 2:** Implementation challenges and strategies for mobile triage units in sub-Saharan Africa

Challenge	Strategies
Financial constraints: High costs ($50 000–$100 000 per MTU); donor dependency	Cost-effective designs (e.g., motorcycle-based MTUs); local manufacturing; public–private partnerships
Workforce Shortages: Lack of trained EMS personnel; 30% turnover in Kenya	Scalable training for CFRs/paramedics; train-the-trainer models; incentives for retention
Logistical Barriers: Poor roads; >2-h delays in Ethiopia during rainy seasons	Motorcycle-based MTUs; infrastructure investments (roads, communication)
Integration issues: Weak referral systems; lack of prehospital notification	Standardized protocols (e.g., SATS); mobile/radio notification systems
Cultural Resistance: Mistrust of CFRs; preference for traditional healers in Malawi	Community engagement; awareness campaigns; involve local leaders
Sustainability: Donor-dependent pilots fail (e.g., Malawi program discontinued)	Policy integration; government funding; regional collaborations

### Training and workforce shortages

SSA faces severe shortages of trained EMS personnel. For example, Malawi has fewer than 200 certified paramedics for a population of over 18 million^[81]^. Task-shifting models, training community health workers or lay first responders to provide basic trauma care, are essential to MTU deployment^[82]^. Advanced skills training requires investment in curriculum development, simulation-based learning, and ongoing mentorship^[83]^. Programs such as STOP THE BLEED have been proposed to scale public trauma care, equipping laypersons with skills to control hemorrhage and provide initial stabilization before MTU arrival^[84]^. Workforce retention is further undermined by brain drain, limiting scalability. In rural Tanzania, fewer than 20% of hospitals have personnel trained to perform emergency laparotomies, highlighting the need for robust prehospital interventions^[58]^.

### Logistical barriers

Geographic isolation, poor road infrastructure, and seasonal weather significantly hinder MTU operations. For instance, over 60% of rural roads in SSA are unpaved, becoming largely impassable during the rainy season^[84]^. Fuel shortages, vehicle maintenance challenges, and limited access to service centers further delay response times^[85]^. These constraints are critical for abdominal trauma patients, where delayed intervention can result in irreversible hemorrhagic shock^[70]^. Innovative solutions such as motorcycle-based MTUs or drone-assisted supply delivery have reduced median transport times by up to 75 min in pilot programs^[67,73]^.

### Integration with existing systems

Successful MTU deployment requires integration into fragmented healthcare networks. Weak communication infrastructure and the absence of standardized referral protocols often delay hospital preparation, prolonging transport times or causing inappropriate referrals^[86,87]^. Implementing interoperable mobile or radio-based communication and prehospital notification systems is essential for optimizing trauma outcomes.

### Cultural and community acceptance

Community trust is vital for MTU’s success. Skepticism toward non-traditional providers, such as community first responders (CFRs), and preference for traditional healers can limit adoption. In Rwanda, involving community leaders and training local volunteers improved uptake of prehospital interventions^[65]^. Education campaigns demonstrating MTU benefits are critical to fostering acceptance in SSA contexts.

### Sustainability

Long-term sustainability depends on robust policy frameworks, government commitment, and local financing mechanisms. Many MTU initiatives remain pilot programs funded by external donors, ceasing when funding ends^[74,76]^. Integrating MTUs into national health budgets, establishing operational standards and licensing frameworks, and aligning with EMS policies are essential to institutionalizing MTUs. Public–private partnerships and multi-stakeholder collaborations can enhance both financial and operational sustainability.

## Strategies for sustainable implementation

### Cost-effective and context-adapted design

Sustainable MTU programs begin with designing units that are affordable, durable, and adaptable to local conditions. Low-cost MTUs constructed from locally sourced materials and modular components reduce dependence on external funding while supporting local economies^[74,76]^. Modular equipment, such as portable ultrasound devices with interchangeable components, simplifies maintenance, reduces downtime, and allows rapid repair using locally available resources. Vehicle-based MTUs optimized for maneuverability can navigate rural roads, unpaved routes, and narrow paths, ensuring timely response in areas with poor infrastructure^[67]^. Collaboration with local manufacturers for MTU components not only enhances affordability but also fosters community engagement and ownership. Evidence from Rwanda and Uganda indicates that cost-effective MTU design increases implementation^[66,67]^.

### Scalable training programs

A competent workforce is central to MTU’s effectiveness. Training programs should target paramedics, CFRs, and emergency medical personnel, emphasizing practical skills for prehospital care in resource-constrained settings, including POCUS, hemorrhage control, airway management, and rapid triage^[8,64,65]^. Scalable strategies include train-the-trainer models, partnerships with academic institutions and NGOs, and blended learning approaches combining in-person and online platforms^[83]^. Certification programs, career incentives (stipends, progression pathways), and ongoing mentorship improve retention and maintain proficiency, particularly for advanced interventions such as prehospital resuscitation and ultrasound-guided diagnostics^[72,73]^.

### Infrastructure and communication enhancements

Robust infrastructure is essential for effective MTU deployment. Investments in road networks, particularly in high-trauma or rural regions, reduce response times and facilitate rapid access to definitive care^[41,42]^. Reliable communication systems enable real-time coordination between MTUs and hospitals, allowing prehospital notification and early mobilization of trauma teams^[87]^. Governments and development partners should integrate these infrastructure improvements into the broader health system, planning to strengthen trauma care delivery.

### Public–private partnerships

Collaborations with NGOs, private companies, and international organizations provide funding, technical expertise, and innovative solutions for MTU programs. Rwanda’s drone delivery program, implemented in partnership with Zipline, reduced delivery times for blood products from 2 to 3 h to 30 min, demonstrating significant benefits for abdominal trauma patients^[79]^. Its success was facilitated by strong government support, dedicated funding, favorable geography for drone operations, and integration with national health systems. Challenges encountered during scaling included regulatory approvals, airspace coordination, and logistical integration with remote health facilities. These factors highlight the importance of contextual enablers and careful planning when considering replication of such programs in other SSA settings. Similar partnerships can support procurement of MTU equipment, vehicle maintenance, training, and technological enhancements, reducing reliance on constrained government budgets. Engagement with private telecommunications providers can further strengthen prehospital communication networks, enable timely hospital notifications, and coordinate trauma responses.

### Community engagement and acceptance

Community trust is crucial for MTU utilization. Awareness campaigns emphasize the life-saving role of MTUs, participatory program design, and inclusion of local leaders in planning enhances legitimacy and overcomes cultural barriers^[65]^. Culturally sensitive education programs in local languages can dispel misconceptions, encourage timely activation of MTU services, and reinforce the importance of rapid trauma care. Evidence from Rwanda and Uganda indicates that engaging communities in MTU design and implementation increases service uptake and improves prehospital outcomes for trauma patients^[66,67]^.

### Policy integration and sustainability

Embedding MTUs within national EMS frameworks ensures long-term sustainability and institutionalization. Governments should establish regulatory standards covering MTU licensing, staffing, equipment specifications, and training certification. Dedicated budget lines for MTU programs within national health plans are essential to secure ongoing funding. South Africa’s integration of MTUs into its Level III trauma system, supported by government funding and standardized protocols, serves as a model for SSA^[62]^. Regional collaborations, such as through the African Federation for Emergency Medicine, can facilitate knowledge exchange, harmonize policies, and support cross-country scaling of MTU programs, enabling broader impact across the continent.

## Limitations

This review has limitations that should be considered when interpreting its findings. The available literature on MTUs in SSA is limited, with most studies consisting of small-scale pilot programs, single-center reports, or descriptive accounts rather than large, multicenter trials. This restricts the generalizability of reported outcomes and limits robust quantitative synthesis. In addition, the heterogeneity of included studies precluded meta-analysis and necessitated a narrative synthesis. Variations in study design, MTU configurations, staffing models, and outcome measures may introduce bias and reduce comparability across contexts. Despite these limitations, this review provides a comprehensive synthesis of current evidence, identifies critical gaps, and offers strategic recommendations for the scalable implementation of MTUs in resource-constrained SSA settings.

## Conclusion

Abdominal trauma remains a major public health challenge in SSA, with high morbidity and mortality driven by systemic deficiencies in prehospital and hospital care. Trauma-related deaths in the prehospital phase may account for up to 50% of mortality, reflecting the absence of organized EMS, reliance on informal transport, and limited hospital resources. MTUs offer a promising approach to address these gaps by providing rapid on-scene triage, stabilization, and diagnostics, potentially improving outcomes for both penetrating and blunt abdominal injuries. Evidence from pilot programs in Uganda, Rwanda, and South Africa suggests that MTUs can reduce prehospital mortality and shorten transport times, enabling timely interventions within the critical “golden hour”.

Innovations such as POCUS, telemedicine, drone-assisted supply delivery, and solar-powered units enhance MTUs’ adaptability to SSA’s resource-constrained and geographically diverse environments. However, financial constraints, workforce shortages, logistical barriers, integration challenges, cultural resistance, and sustainability issues remain critical obstacles to widespread implementation. Addressing these challenges requires a multi-pronged approach, including cost-effective and locally adaptable MTU designs, scalable training programs, public–private partnerships, community engagement, and integration into national EMS frameworks. Future research should focus on larger, multicenter evaluations to quantify MTU effectiveness, optimize operational protocols, and identify best practices for sustainable deployment across SSA.

## Data Availability

Data sharing is not applicable to this article as no datasets were collected.
